# LAT1 and SNAT2 Protein Expression and Membrane Localization of LAT1 Are Not Acutely Altered by Dietary Amino Acids or Resistance Exercise Nor Positively Associated with Leucine or Phenylalanine Incorporation in Human Skeletal Muscle †

**DOI:** 10.3390/nu13113906

**Published:** 2021-10-30

**Authors:** Michael Mazzulla, Nathan Hodson, Matthew Lees, Paula J. Scaife, Kenneth Smith, Philip J. Atherton, Dinesh Kumbhare, Daniel R. Moore

**Affiliations:** 1 Department of Exercise Sciences, Faculty of Kinesiology and Physical Education, University of Toronto, Toronto, ON M5S 2C9, Canada; m.mazzulla@mail.utoronto.ca (M.M.); nathan.hodson@utoronto.ca (N.H.); matthew.lees@utoronto.ca (M.L.); 2 MRC-Versus Arthritis Centre for Musculoskeletal Ageing Research and NIHR Nottingham BRC, Centre of Metabolism, Ageing and Physiology, School of Medicine, University of Nottingham, Derby DE22 3DT, UK; Paula.Scaife@nottingham.ac.uk (P.J.S.); ken.smith@nottingham.ac.uk (K.S.); philip.atherton@nottingham.ac.uk (P.J.A.); 3 Department of Medicine, University of Toronto, Toronto, ON M5S 2C9, Canada; dinesh.kumbhare@uhn.ca

**Keywords:** dietary protein, resistance exercise, protein, skeletal muscle, amino acid transporters

## Abstract

The influx of essential amino acids into skeletal muscle is primarily mediated by the large neutral amino acid transporter 1 (LAT1), which is dependent on the glutamine gradient generated by the sodium-dependent neutral amino acid transporter 2 (SNAT2). The protein expression and membrane localization of LAT1 may be influenced by amino acid ingestion and/or resistance exercise, although its acute influence on dietary amino acid incorporation into skeletal muscle protein has not been investigated. In a group design, healthy males consumed a mixed carbohydrate (0.75 g·kg^−1^) crystalline amino acid (0.25 g·kg^−1^) beverage enriched to 25% and 30% with LAT1 substrates L-[1-^13^C]leucine (LEU) and L-[*ring*-^2^H_5_]phenylalanine (PHE), respectively, at rest (FED: *n* = 7, 23 ± 5 y, 77 ± 4 kg) or after a bout of resistance exercise (EXFED: *n* = 7, 22 ± 2 y, 78 ± 11 kg). Postprandial muscle biopsies were collected at 0, 120, and 300 min to measure transporter protein expression (immunoblot), LAT1 membrane localization (immunofluorescence), and dietary amino acid incorporation into myofibrillar protein (ΔLEU and ΔPHE). Basal LAT1 and SNAT2 protein contents were correlated with each other (*r* = 0.55, *p* = 0.04) but their expression did not change across time in FED or EXFED (all, *p* > 0.05). Membrane localization of LAT1 did not change across time in FED or EXFED whether measured as outer 1.5 µm intensity or membrane-to-fiber ratio (all, *p* > 0.05). Basal SNAT2 protein expression was not correlated with ΔLEU or ΔPHE (all, *p* ≥ 0.05) whereas basal LAT1 expression was negatively correlated with ΔPHE in FED (*r* = −0.76, *p* = 0.04) and EXFED (*r* = −0.81, *p* = 0.03) but not ΔLEU (*p* > 0.05). Basal LAT1 membrane localization was not correlated with ΔLEU or ΔPHE (all, *p* > 0.05). Our results suggest that LAT1/SNAT2 protein expression and LAT1 membrane localization are not influenced by acute anabolic stimuli and do not positively influence the incorporation of dietary amino acids for de novo myofibrillar protein synthesis in healthy young males.

## 1. Introduction

Exogenous amino acid administration increases the uptake of amino acids into skeletal muscle at rest and, to a greater extent, after resistance exercise [1]. Dietary amino acids, in particular the essential amino acids, are prime stimulators of skeletal muscle anabolism [2] and have been suggested to be preferential substrates for muscle protein synthesis after exposure to anabolic stimuli [3,4]. Muscle protein synthesis rates are stimulated within 2 h post-feeding/exercise with rapidly digested amino acid sources [5,6,7] and are underpinned by increases in extracellular and intracellular essential amino acid availability within 30 min of amino acid intake [1,2]. The ability of dietary amino acids to stimulate muscle protein synthesis is generally dependent on their delivery to intracellular sensors and effector molecules associated with the mTORC1 pathway [8] of which amino acid transporters at the plasma membrane are an essential but not rate limiting component [9,10]. The influx of essential amino acids into skeletal muscle is primarily mediated by the large neutral amino acid transporter 1 (LAT1), which is dependent on the glutamine gradient generated by the sodium-dependent neutral amino acid transporter 2 (SNAT2) [11,12]. LAT1 is the most highly expressed large neutral amino acid transporter in skeletal muscle [13], although others such as LAT2, LAT3, and LAT4 [14,15] may be present and able to transport essential amino acids. Importantly, however, in skeletal muscle lacking LAT1 expression, phenylalanine uptake into skeletal muscle is drastically reduced, while intramuscular leucine concentrations in response to intraperitoneal leucine administration remained unchanged [13]. As such, LAT1 seems to be the primary contributor to both leucine and phenylalanine influx into skeletal muscle. In addition to their role in inward amino acid transport, membrane-bound transporters have been suggested to have auxiliary roles as extracellular amino acid sensors [16,17,18]. Thus, amino acid transporters appear to play an integral role in the anabolic response to dietary amino acids and exercise, although the relationship between the protein expression and membrane content of key amino acid transporters, and the subsequent incorporation of dietary amino acids to support de novo protein synthesis, is lacking.

There is evidence to suggest that, similar to muscle protein synthesis rates, amino acid transporter expression in human muscle may be dynamic and acutely responsive to dietary amino acids [19] and resistance exercise [20], which suggests these transporters may have a role in the regulation of acute protein synthetic events. For example, amino acid ingestion stimulates an increase in LAT1 and SNAT2 gene expression, an event which is followed by increases in protein expression 3 h post-feeding [19]. These changes have also been reported in response to resistance exercise alone [20] and after the combination of feeding and resistance exercise [21], whereby LAT1 and SNAT2 gene expression increase 6 h post-exercise followed by increases in protein expression over a 24-h recovery period. Changes in LAT1 and SNAT2 transporter expression have also been observed in isolated membrane fractions during periods of low amino acid availability following acute resistance exercise [22]. Collectively, these data suggest that feeding and resistance exercise influence the gene and protein expression of LAT1 and SNAT2 that could contribute to enhanced amino acid transport and a greater protein synthetic response within skeletal muscle [1,2,8], although we [23] and others [24] have failed to replicate these acute protein expression changes. However, extracellular essential amino acid availability [1,2] and the inward transport of amino acids in skeletal muscle [25] is increased early after feeding and, as a result, muscle protein synthesis rates are stimulated within 2 h post-feeding/exercise [5,6,7]. Furthermore, LAT1 must be associated with the sarcolemmal membrane to be active and carry free amino acids into muscle cells [26]. Thus, it is unclear whether previously reported increases in gene and/or protein expression without insight into the cellular location of LAT1 influence the acute postprandial protein synthetic response and, more importantly, the incorporation of dietary amino acids for muscle protein remodeling.

Therefore, the purpose of this study was to determine whether LAT1 and SNAT2 protein expression and the membrane localization of LAT1 are influenced by the anabolic effect of amino acid ingestion at rest and after resistance exercise. Importantly, we provided a complete amino acid beverage labeled with L-[1-^13^C]leucine and L-[*ring*-^2^H_5_]phenylalanine to assess the physiological role for these transporters in the incorporation of dietary amino acids for de novo muscle protein synthesis. We utilized a dual oral tracer model, as previous studies measuring dietary amino acid incorporation after feeding and resistance exercise have only traced phenylalanine into skeletal muscle [5,6,7], which would represent only one substrate (leucine being another) for LAT1 [27]. Furthermore, there is evidence to suggest that leucine and phenylalanine are equivalent substrates for LAT1 [28] but that there may be two pools of muscle intracellular leucine whereby exogenous leucine is primarily directed toward oxidation, and intracellular leucine from protein breakdown is preferentially reutilized for protein synthesis [29]. This influenced our decision to include two metabolic tracers of different essential amino acids, one of which (leucine) is generally considered to be the preeminent anabolic amino acid [11,30,31]. We hypothesized that, in line with our previous findings [23], amino acid ingestion at rest and after resistance exercise would not increase LAT1 and SNAT2 protein expression nor LAT1 membrane localization in the early postprandial period but that there would be a positive association between basal transporter expression/membrane localization and dietary amino acid incorporation into myofibrillar protein.

## 2. Materials and Methods

### 2.1. Participants

Fourteen healthy males were included in the study (Characteristics in Table 1). Participants were recruited via postings at the University of Toronto and were recreationally active (e.g., performed weightlifting, running, team-sport activity) 2–5 times per week for at least six months before enrolment. Participants were considered healthy based on responses to the PAR-Q+ and a medical history form. The data presented herein are part of a secondary analysis to the primary outcome of a larger study, which was registered as a Clinical Trial at ClinicalTrials.gov (protocol code NCT04887727).

### 2.2. Experimental Design

A group design was used in the present study. Participants reported to the laboratory for baseline testing 5–7 d before the metabolic trial. Participants were familiarized with the whole-body resistance exercise protocol and underwent 1-repitition maximum (1-RM) testing for the following exercises: (i) dumbbell bench press; (ii) dumbbell bent over row; (iii) leg press; and (iv) leg extension. After baseline testing participants were randomly assigned to a rested-fed (FED) or exercise-fed (EXFED) condition. Participants were instructed to refrain from alcohol/caffeine consumption and vigorous exercise for 48 h before the metabolic trial and to consume their typical diet. Dietary intake was analyzed for energy and macronutrient content using The Food Processor^®^ Nutrition Analysis Software (ESHA, Salem, OR, USA).

### 2.3. Metabolic Trial

Participants reported to the laboratory at ~0700 h after an overnight fast for the metabolic trial. Participants rested in a supine position and a baseline skeletal muscle biopsy (PRE) was obtained from the middle region of the vastus lateralis using a 5 mm Bergström needle modified for manual suction [32] under 2% lidocaine local anesthesia to determine background enrichments of L-[1-^13^C]leucine and L-[*ring*-^2^H_5_]phenylalanine and basal LAT1/SNAT protein expression. Muscle samples were freed from visible blood, fat, and connective tissue, rapidly frozen in liquid nitrogen, and stored at −80 °C until further analysis. Immediately after this biopsy, participants in EXFED performed a bout of whole-body resistance exercise consisting of 4 × 10 repetitions each of dumbbell bench press, dumbbell bent over row, leg press, and leg extension, while participants in FED rested. Each exercise was performed at 75% of their pre-determined 1-RM with ~90 s rest between sets. Whole-body resistance exercise was selected as the exercise stimulus for the measurement of whole-body amino acid kinetics (not presented here) and to provide ecological validity for exercised individuals included in the study. Immediately after cessation of exercise, all participants ingested a beverage containing 0.25 g·kg^−1^ protein as crystalline amino acids (Ajinomoto Co., Inc., Raleigh, NC, USA) modeled on the composition of egg protein [33] with 0.75 g·kg^−1^ carbohydrate (TANG, Kraft Canada Inc., Mississauga, ON, Canada) dissolved in 500 mL water. The leucine and phenylalanine content of the beverage was enriched to 25% and 30% with L-[1-^13^C]leucine (99% at, CIL Canada Inc., Montreal, PQ, Canada) and L-[*ring*-^2^H_5_]phenylalanine (99% at, CIL Canada Inc.), respectively. This enrichment level is similar to what we [34] and others [35] have obtained with intrinsically-labeled proteins and is suitable to detect changes in tracer-to-tracee ratio (TTR) within skeletal muscle. Subsequent muscle biopsies were obtained from separate incisions (~2–3 cm apart) in alternating legs at *t* = 120 and 300 min after beverage ingestion to measure time-course changes in LAT1 and SNAT2 protein expression and LAT1 membrane content. Changes in dietary amino acid incorporation were determined from the TTR of L-[1-^13^C]leucine (ΔLEU) and L-[*ring*-^2^H_5_]phenylalanine (ΔPHE) over the entire postprandial period using the 300-min biopsy [36].

### 2.4. Skeletal Muscle Analyses

Myofibrillar protein-enriched fractions were isolated from ~25 mg wet muscle tissue in the Iovate/MuscleTech Metabolism and Sports Science Lab at the University of Toronto as previously described [23]. Myofibrillar-bound protein enrichments of L-[1-^13^C]leucine were determined by gas chromatography-combustion-isotope ratio mass spectrometry [37] and L-[*ring*-^2^H_5_]phenylalanine enrichments were determined by liquid chromatography-tandem mass spectrometry [23]. Immunoblotting procedures were conducted according to previously described methods [23,38]. Antibodies utilized were as follows: LAT1 (ab85226) and SNAT2 (ab90677) purchased from Abcam (Toronto, Canada) and diluted in 5% BSA (1:1000). Values of basal LAT1 and SNAT2 protein expression were determined by placing raw band intensity values in relation to the corresponding Ponceau value. LAT1 visualization via immunofluorescence microscopy was conducted as described previously by our group with dystrophin demarking the cell membrane [38]. Membrane localization of LAT1 was then quantified as LAT1 signal intensity within the outer 1.5 µm of fibers and membrane-to-fiber ratio was calculated as ‘membrane’ LAT1 signal intensity expressed in relation to LAT1 signal intensity in the remainder of each fiber. All immunofluorescent image analysis was conducted on ImageJ software (Fiji plugin, v. 1.5, National Institutes of Health, Bethesda, MD, USA).

### 2.5. Statistical Analyses

Statistical analyses were performed on SPSS Statistics (Version 26, IBM, Armonk, NY, USA). Differences in amino acid transporter expression and LAT1 membrane localization/membrane-to-fiber ratio were tested using a mixed-design two-factor ANOVA with time as the within-subject factor and condition as the between-subject factor. Where sphericity was violated a Greenhouse–Geisser correction was applied to all main effects and interactions, and if data were not normally distributed, logarithmic transformations were conducted. Where significant interactions were identified in the ANOVA a Bonferroni post hoc test was performed to determine differences between means for all significant main effects and interactions. An unpaired t test was used to test differences in dietary amino acid incorporation. Simple linear regression was applied to test for correlations between amino acid incorporation and basal LAT1/SNAT2 protein expression and LAT1 membrane localization. For all analyses, the level of significance was *p* < 0.05. Results are presented as the means ± SD.

## 3. Results

### 3.1. Participant Characteristics

There were no differences (all comparisons, *p* > 0.05) between conditions with respect to participants’ baseline characteristics (Table 1).

### 3.2. Amino Acid Transporter Expression

**Figure 1:**
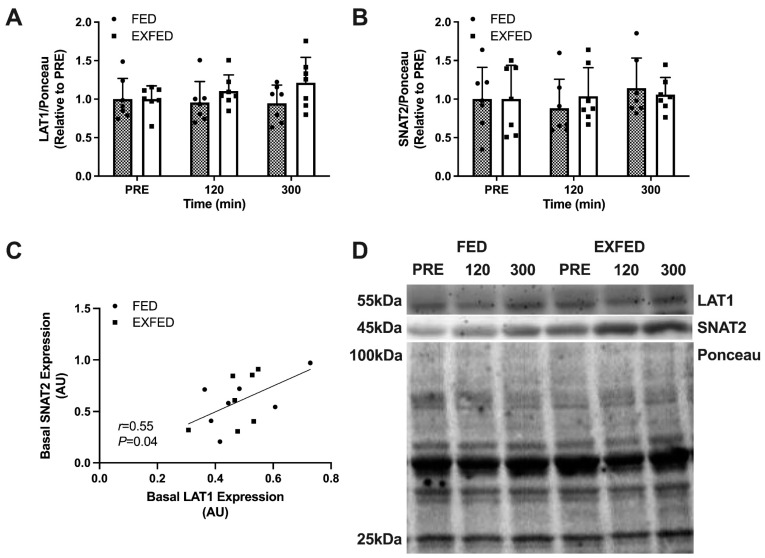
Amino acid transporter expression: (**A**) LAT1/Ponceau protein expression; (**B**) SNAT2/Ponceau protein expression; (**C**) Basal LAT1 protein expression vs. basal SNAT2 protein expression; and (**D**) Representative blots for each target. PRE: baseline muscle sample. Data are the means ± SD. FED: *n* = 7; EXFED: *n* = 7. Differences in amino acid transporter expression were tested using a mixed-design two-factor ANOVA with time as the within-subject factor and condition as the between-subject factor: all comparisons, *p* > 0.05.

Protein expression of LAT1 and SNAT2 (Figure 1A,B) were not influenced by amino acid ingestion at rest or after exercise. Specifically, protein expression of LAT1 (Figure 1A) and SNAT2 (Figure 1B) did not differ across time points (LAT1, time effect: *p* = 0.60; SNAT2, time effect: *p* = 0.50) and there were no differences between conditions (LAT1, condition effect: *p* = 0.13; SNAT2, condition effect: *p* = 0.84) at any time point (LAT1, interaction effect: *p* = 0.33; SNAT2, interaction effect: *p* = 0.58). When collapsed across conditions, basal LAT1 protein content was positively correlated (*r* = 0.55, *p* = 0.04) with basal SNAT2 protein content (Figure 1C).

### 3.3. LAT1 Membrane Localization

**Figure 2:**
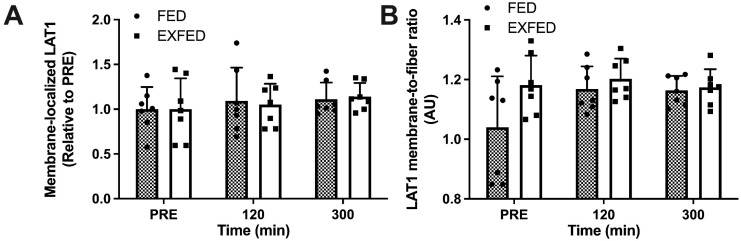

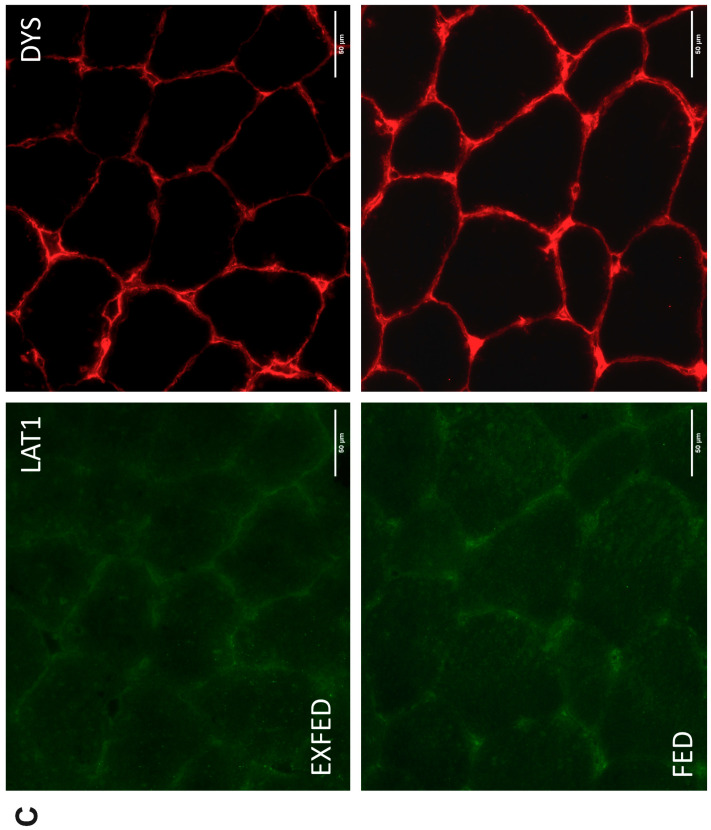
LAT1 membrane localization: (**A**) Membrane-localized LAT1; (**B**) LAT1 membrane-to-fiber ratio; and (**C**) Representative stain for membrane-localized LAT1. PRE: baseline muscle sample. Data are the means ± SD. FED: *n* = 7; EXFED: *n* = 7. Differences in LAT1 membrane content were tested using a mixed-design two-factor ANOVA with time as the within-subject factor and condition as the between-subject factor: all comparisons, *p* > 0.05.

Membrane localization of LAT1 did not change in response to amino acid ingestion at rest or after exercise when measured as outer 1.5 µm intensity (Figure 2A) or membrane-to-fiber ratio (Figure 2B). Specifically, LAT1 membrane content when measured as outer 1.5 µm intensity did not differ across time points (time effect: *p* = 0.40) and there were no differences between conditions (condition effect: *p* = 0.96) at any time point (interaction effect: *p* = 0.93). When measured as membrane-to-fiber ratio, LAT1 membrane content did not differ across time points (time effect: *p* = 0.08) and there were no differences between conditions (condition effect: *p* = 0.13) at any time point (interaction effect: *p* = 0.12) (Figure 2C).

### 3.4. Dietary Amino Acid Incorporation

**Figure 3:**
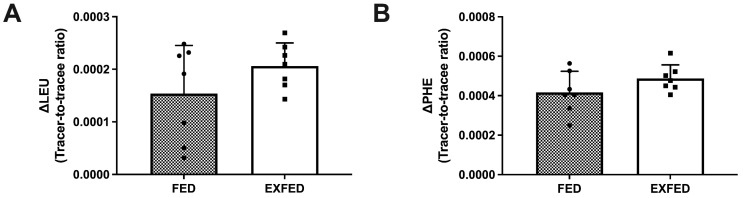
Dietary amino acid incorporation: (**A**) Myofibrillar protein-bound enrichment representing dietary incorporation of L-[1-^13^C]leucine (ΔLEU); (**B**) Myofibrillar protein-bound enrichment representing dietary incorporation of L-[*ring*-^2^H_5_]phenylalanine (ΔPHE). Data are the means ± SD with scatter plot of individual data points. FED: *n* = 7; EXFED: *n* = 7. An unpaired t test was used to test differences in dietary amino acid incorporation: *p* > 0.05.

Resistance exercise did not influence dietary amino acid incorporation. Specifically, myofibrillar protein-bound TTR representing dietary incorporation of L-[1-^13^C]leucine (ΔLEU; Figure 3A) did not differ (*p* = 0.20) between conditions. Myofibrillar protein-bound TTR representing dietary incorporation of L-[*ring*-^2^H_5_]phenylalanine (ΔPHE; Figure 3B) did not differ (*p* = 0.16) between conditions.

### 3.5. Correlations

**Figure 4:**
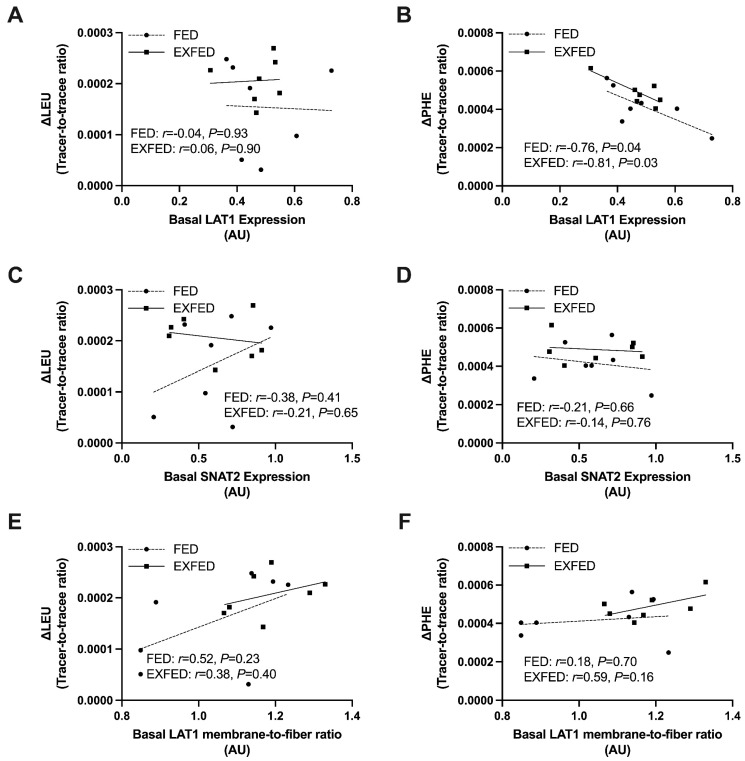
Correlations between basal transporter expression/LAT1 membrane-to-fiber ratio and dietary amino acid incorporation: (**A**) Basal LAT1 protein expression vs. ΔLEU; (**B**) Basal LAT1 protein expression vs. ΔPHE; (**C**) Basal SNAT2 protein expression vs. ΔLEU; (**D**) Basal SNAT2 protein expression vs. ΔPHE; (**E**) Basal LAT1 membrane-to-fiber ratio vs. ΔLEU; (**F**) Basal LAT1 membrane-to-fiber ratio vs. ΔPHE. FED (dotted line): *n* = 7; EXFED (solid line): *n* = 7. Simple linear regression was applied to test for correlations between amino acid incorporation and basal LAT1/SNAT2 protein expression.

There was no association between basal LAT1 protein expression and dietary amino acid incorporation assessed by L-[1-^13^C]leucine (Figure 4A) in FED (*r* = −0.04, *p* = 0.93) or EXFED (*r* = 0.05, *p* = 0.90). However, there was a negative correlation between basal LAT1 protein expression and dietary amino acid incorporation assessed by L-[*ring*-^2^H_5_]phenylalanine (Figure 4B) in FED (*r* = −0.76, *p* = 0.04) and EXFED (*r* = −0.81, *p* = 0.03). There was no association between basal SNAT2 protein expression and dietary amino acid incorporation assessed by L-[1-^13^C]leucine (Figure 4C) in FED (*r* = 0.37, *p* = 0.41) or EXFED (*r* = −0.21, *p* = 0.65). There was no association between basal SNAT2 protein content and dietary amino acid incorporation assessed by L-[*ring*-^2^H_5_]phenylalanine (Figure 4D) in FED (*r* = −0.21, *p* = 0.66) or EXFED (*r* = −0.14, *p* = 0.76). There was no association between basal LAT1 membrane-to-fiber ratio and dietary amino acid incorporation assessed by L-[1-^13^C]leucine (Figure 4E) in FED (*r* = 0.52, *p* = 0.23) or EXFED (*r* = 0.38, *p* = 0.40). There was no association between basal LAT1 membrane-to-fiber ratio and dietary amino acid incorporation assessed by L-[*ring*-^2^H_5_]phenylalanine (Figure 4F) in FED (*r* = 0.18, *p* = 0.70) or EXFED (*r* = 0.59, *p* = 0.16).

## 4. Discussion

The incorporation of exogenous amino acids, in particular the essential amino acids, into skeletal muscle is facilitated by amino acid transporters LAT1 and SNAT2 [12]. These transporters work in tandem to transport dietary leucine and phenylalanine into the cell via a bi-transport system which simultaneously exports glutamine and histidine [10]. LAT1 forms a heterodimeric amino acid transporter with ancillary glycoproteins and catalyzes the substrate transporting capacity of the transport complex [39]. Thus, unsurprisingly, basal LAT1 and SNAT2 protein expression were positively correlated in the present study, which likely reflects the inter-dependence of LAT1 on SNAT2 for the transport, uptake, and incorporation of essential amino acids such as leucine and phenylalanine into skeletal muscle [9]. It is noteworthy that both feeding and resistance exercise increase the rates of tracer-derived uptake of SNAT2 substrates (e.g., alanine, glutamine) in conjunction with LAT1 substrates leucine and phenylalanine [1,40,41]. However, in our study, LAT1 and SNAT2 protein expression did not change in response to amino acid ingestion at rest or after resistance exercise, nor did the membrane localization of LAT1 change when measured as outer 1.5 µm intensity or membrane-to-fiber ratio. These results are in contrast to previous studies showing an increase in LAT1 and SNAT2 protein expression after both feeding [19] and resistance exercise [20], but consistent with our previous work [23] and that of others [24] who have observed no change in amino acid transporter protein expression following acute exposure to anabolic stimuli. Thus, while limited in sample size, the present results do not support a role for anabolic stimuli to alter the protein expression of these amino acid transporters, nor the membrane localization of LAT1 as a means to increase amino acid-induced muscle protein anabolism during the acute postprandial period.

Amino acid feeding at rest, and to a greater extent after resistance exercise, enhances muscle protein synthesis rates via increased amino acid uptake into skeletal muscle [1]. Indeed, it has been suggested that exogenous amino acids may be used as a preferential substrate for stimulating muscle protein synthesis rates after exposure to anabolic stimuli [3,4]. In contrast to our hypothesis, we did not observe a positive association between basal SNAT2 protein expression and dietary leucine or phenylalanine incorporation into skeletal muscle after amino acid ingestion at rest and after resistance exercise. Although the antiporter LAT1 requires intracellular glutamine for inward essential amino acid transport, glutamine is the most abundant amino acid in skeletal muscle (~20 mM) and its inward transport substantially increases in response to exogenous amino acids and insulin [41,42], suggesting its intracellular concentration is not rate-limiting for essential amino acid uptake in otherwise healthy individuals. In contrast, the basal expression of LAT1 did not influence dietary leucine incorporation, but was negatively correlated with phenylalanine incorporation into myofibrillar protein. These disparate findings between presumably equivalent LAT1 substrates [28] may be explained in part by the potential for separate intracellular leucine pools that favor the oxidation of externally derived amino acids [29]. In contrast, phenylalanine is oxidized in the liver [43] and would only be utilized for protein synthesis within skeletal muscle, which may explain our ability to delineate a correlation (albeit negative) between dietary phenylalanine incorporation and LAT1 expression. However, it should be noted that LAT1 is a bidirectional transporter and can coordinate both the influx and efflux of amino acids from skeletal muscle, both of which are enhanced in response to exogenous amino acids at rest and after exercise [1,44]. Therefore, it is possible that a higher basal LAT1 expression in skeletal muscle may promote a greater phenylalanine transmembrane flux, which could reduce the ability of exogenous phenylalanine to be utilized for de novo protein synthesis.

Dietary amino acids were incorporated into skeletal muscle without a concomitant increase in amino acid transporter expression in the present study, which may be explained in part by the dissociation between amino acid transporter expression and location/activity [45]. For example, treatment of skeletal muscle cells with insulin increases SNAT2 activity and recruitment into the plasma membrane [46] which, if this occurred in the present study, our mixed muscle lysate would preclude our ability to detect. However, LAT1 is the principal transporter of leucine and phenylalanine into muscle cells [27] and must be associated with the sarcolemmal membrane in order to be active and carry free leucine (and phenylalanine) into muscle cells [26]. LAT1 is in close proximity to capillaries in young skeletal muscle [38] which may suggest that peripheral localization of mTOR within the muscle cell, which we observed in response to anabolic stimuli [47,48], could position it in closer proximity to amino acid substrates of LAT1 (e.g., ingested leucine and phenylalanine). Post-translational modifications and/or altered membrane expression of LAT1 and SNAT2 can also occur in response to anabolic stimuli [22,49], which may ultimately be more influential for the regulation of dietary amino acid incorporation into human skeletal muscle. However, we did not observe alterations in the membrane localization of LAT1 in response to feeding and rest or feeding after resistance exercise, which is supported by findings from Agergaard and colleagues [22] who showed that LAT1 expression in isolated membrane fractions did not change in response to bolus protein ingestion but only during periods of low amino acid availability following placebo and pulse feeding. This could suggest that membrane bound LAT1 increases only when large amounts of exogenous amino acids are not readily available to muscle fibers. Furthermore, although chronic resistance exercise was shown to elevate LAT1 protein content in whole muscle lysates, membrane associated LAT1 was not affected [50]. As such, available evidence suggests that membrane associated/localized LAT1 is unaffected by both acute and chronic anabolic stimuli. LAT1 must also form a heterodimer with 4F2hc/CD98(SLC3A2) in order to localize to membranes and be optimally active [51] so it is possible that changes in localization/expression of this support protein may also be implicated in skeletal muscle dietary amino acid incorporation. However, previous data in human skeletal muscle have failed to observe changes in 4F2hc protein expression following essential amino acid feeding [19] or lower-body resistance exercise [20]. As such, we hypothesize 4F2hc protein expression would also remain unchanged in the current study. Alternatively, the inside-out stimulation of mTORC1 by leucine and other essential amino acids via V-ATPases has been suggested to require the recruitment of LAT1 to the lysosomal membrane [52], which would align with our previous observations of the lysosome being a focal point of mTORC1 activation with feeding and exercise in human muscle [48,53,54].

## 5. Conclusions

In conclusion, we demonstrated that, despite basal correlation in protein expression, amino acid ingestion at rest and after resistance exercise did not increase the expression of skeletal muscle amino acid transporters LAT1 and SNAT2, nor did it increase the membrane localization of LAT1. Moreover, despite the ability of exogenous amino acids to stimulate acute muscle protein anabolism, basal amino acid transporter protein expression and LAT1 membrane localization do not positively influence the incorporation of essential amino acids leucine and phenylalanine after feeding and resistance exercise. Thus, despite suggestions that dietary amino acids represent primary substrates for skeletal muscle anabolism (i.e., muscle protein synthesis), our results suggest that this process is not dependent on the basal expression of amino acid transporters nor the membrane localization of LAT1 when characterized over the acute postprandial period.

## Figures and Tables

**Table 1:** Participant characteristics ^1^.

Characteristic	FED (*n* = 7)	EXFED (*n* = 7)
Age, y	23 ± 5	22 ± 2
Height, cm	179 ± 5	177 ± 9
Body mass, kg	77 ± 4	78 ± 11
Fat-free mass ^2^, kg	66 ± 4	69 ± 11
Body fat, %	14 ± 4	12 ± 5
Habitual dietary intake ^3^		
Protein, g·kg^−1^·d^−1^	1.8 ± 0.5	1.9 ± 0.6
Carbohydrate, g·kg^−1^·d^−1^	3.4 ± 1.1	4.0 ± 1.6
Fat, g·kg^−1^·d^−1^	1.5 ± 0.8	1.1 ± 0.3

^1^ Values are means ± SD; all comparisons, *p* > 0.05. ^2^ Fat-free mass measured via BOD POD (COSMED USA Inc., Chicago, IL, USA). ^3^ Habitual dietary intake based on 48h diet record analysis (ESHA).

## Data Availability

The data presented in this study are available on request from the corresponding author. The data are not publicly available due to ethical constraints.
